# Carbon allocation across dominance classes varies with planting density, site quality, and climatic moisture deficit in *Cunninghamia lanceolata* plantations

**DOI:** 10.3389/fpls.2026.1878250

**Published:** 2026-07-15

**Authors:** Ziqing Lv, Raphaël Trouvé, Craig R. Nitschke, Xiaoyan Li, Aiguo Duan, Jianguo Zhang

**Affiliations:** 1 State Key Laboratory of Efficient Production of Forest Resources, Key Laboratory of Forest Cultivation and Management of National Forestry and Grassland Administration, Research Institute of Forestry, Chinese Academy of Forestry, Beijing, China; 2 School of Agriculture, Food and Ecosystem Sciences, The University of Melbourne, Melbourne, VIC, Australia; 3 Collaborative Innovation Center of Sustainable Forestry in Southern China, Nanjing Forestry University, Nanjing, China

**Keywords:** carbon allocation, Chinese fir, dominance class, planting density, site index

## Abstract

Chinese fir plantations are a major component of China’s forest carbon sink, and their mitigation value depends not only on total biomass but also on how carbon is allocated among stems, crowns, and roots at the tree level. Carbon allocation responds to competition and resource availability, proxied by stand structure (initial planting density and dominance classes) and environmental conditions (site index and climate). Yet how planting density, site index, and climate are associated with allocation variation across dominance classes remains poorly quantified, limiting density- and site-adaptive management for carbon sequestration. We used long-term density experiments at three subtropical sites (Sichuan, Guangxi, and Fujian), comprising 45 plots and five initial planting densities (1,667 to 10,000 trees ha^-1^). Trees were classified into four dominance classes, organ biomass and carbon were estimated with region-specific additive equations, and the effects of planting density, site index, climatic moisture deficit, and dominance class were evaluated with dominance-class regressions and linear mixed-effects models. From dominant to suppressed trees, the stem biomass fraction increased from 73.1% to 76.8%, whereas branch and root fractions decreased from 6.6% to 4.8% and from 15.0% to 13.1%, respectively. Higher site index was associated with greater stem allocation (+0.33 to +0.59 percentage points per meter) and lower leaf allocation (−0.28 to −0.44 percentage points per meter), whereas stronger competition (indexed by planting density and stand density index) was associated with higher stem but lower branch, leaf, and root allocation. Responses to climatic moisture deficit were dominance-dependent: dominant trees maintained relatively stable root allocation, whereas suppressed trees shifted carbon toward stems and away from roots. These results indicate that competitive structure was more closely associated with estimated carbon-allocation patterns than any single environmental gradient, and that limiting severe suppression and protecting root allocation through density management may help sustain the size and resilience of plantation carbon stocks.

## 1 Introduction

Forests are the largest terrestrial carbon pool and play a key role in removing carbon dioxide from the atmosphere, which can help mitigate global climate change (FAO, 2020; Lewis et al., 2019). Plantation forests, owing to their fast growth and high yield, together with management flexibility, have become a key component of climate change mitigation strategies (Canadell and Raupach, 2008; Piao et al., 2009). To understand how plantations can support China’s goals of achieving peak emissions by 2030 and carbon neutrality by 2060, it is essential to evaluate the carbon sequestration capacity of major plantation species, particularly given that China has the largest plantation area globally (Cheng et al., 2024; Ke et al., 2023).

Forest carbon sequestration depends not only on total biomass stored in trees but also on how carbon is allocated among stems, branches, leaves, and roots, which differ in residence time (Litton et al., 2007; Merganičová et al., 2019). Biomass allocation reflects how trees adapt to the environment and resource competition, with implications for stand stability and productivity, as well as long-term carbon storage (Litton et al., 2007; Poorter et al., 2012; Poorter and Nagel, 2000). Optimal partitioning theory and the functional equilibrium concept predict that plants allocate proportionally more biomass to organs that acquire the most limiting resource (Bloom et al., 1985; Chapin et al., 1987; Poorter et al., 2012). In mature plantations, organ-specific biomass and carbon fractions provide practical structural indicators of longer-term allocation patterns, although they should not be interpreted as direct measurements of instantaneous carbon fluxes (Litton et al., 2007; Poorter et al., 2012). These fractions can vary with tree social status, competition, and site quality (Naidu et al., 1998; Gargaglione et al., 2010).

In forest plantations, resource limitation is strongly modulated by stand density and site conditions: higher tree densities intensify intraspecific competition, reducing light availability and soil moisture, whereas fertile or moist sites can ameliorate below-ground constraints on growth (Ni et al., 2022; Rubio-Cuadrado et al., 2024; Wang et al., 2022). Denser stands promote greater investment in height growth and produce more slender stem forms, as trees prioritize vertical extension to escape shading and maintain competitiveness (Poorter et al., 2012; Trouvé et al., 2025). Several studies in even-aged plantations have reported that increasing stand density shifts biomass allocation toward stems, with stem mass fractions increasing while allocation to branches or foliage declines (Fang et al., 2018; Harris, 2007; Jiao et al., 2022; Wertz et al., 2020). Soil fertility influences resource availability, with trees on richer sites typically allocating more resources to aboveground organs (Deng et al., 2023; Gargaglione et al., 2010; Poorter and Nagel, 2000). Climatic factors further influence carbon allocation by altering soil nutrient availability and leaf functional traits (Gong et al., 2023). Plants in arid or cold environments often prioritize root allocation to enhance resource acquisition (Qi et al., 2019), while global temperature gradients drive broader latitudinal patterns in carbon distribution among organs (Reich et al., 2014). However, few studies have evaluated density, site quality, and climate together when examining biomass and carbon allocation in mature Chinese fir plantations. This makes it difficult to determine whether density effects are stable across environments or vary with site and climatic conditions.

Chinese fir (*Cunninghamia lanceolata* (Lamb.) Hook.) is a native conifer widely distributed in subtropical China and is one of the most important plantation species in the country. According to the ninth National Forest Inventory, Chinese fir plantations cover 9.90 million ha and have a standing stock volume of 756 million m^3^, accounting for 17.33% and 22.30% of China’s arbor-plantation area and stock volume, respectively (National Forestry and Grassland Administration, 2019). These figures make Chinese fir the nation’s leading timber species and an important component of national carbon sequestration efforts. Although Chinese fir plantations are often managed as even-aged monocultures, asymmetric competition can generate tree-size hierarchies and dominance classes (Schwinning and Weiner, 1998). Such structural heterogeneity may influence organ-level biomass and carbon allocation (Naidu et al., 1998; Gargaglione et al., 2010), yet whether allocation responses to planting density, site quality, and climatic moisture deficit differ among dominance classes remains unclear in mature Chinese fir plantations.

In this study, we used long-term Chinese fir planting density experiments established in the early 1980s at three sites in subtropical China, with five initial planting densities at each site. To characterize organ-level biomass and carbon allocation, we combined detailed tree inventory data with region-specific additive equations calibrated from destructive samples collected in the buffer rows of the same experimental system. Trees were classified into four dominance classes, and locally estimated organ biomass and carbon allocation fractions were analyzed across structural and environmental gradients. Our aims were to (1) characterize variation in allometry-derived organ biomass and carbon allocation indicators among dominance classes in mature Chinese fir plantations; and (2) test how planting density, site index, and climatic moisture deficit are associated with these estimated allocation indicators across dominance classes. By explicitly accounting for the local allometric basis and hierarchical stand structure, this study uses long-term density trials to quantify how planting density, site conditions, and climate are associated with estimated organ-level allocation patterns in mature Chinese fir plantations.

## 2 Materials and methods

### 2.1 Study area and forest plot data

#### 2.1.1 Study area description

**Figure 1:**
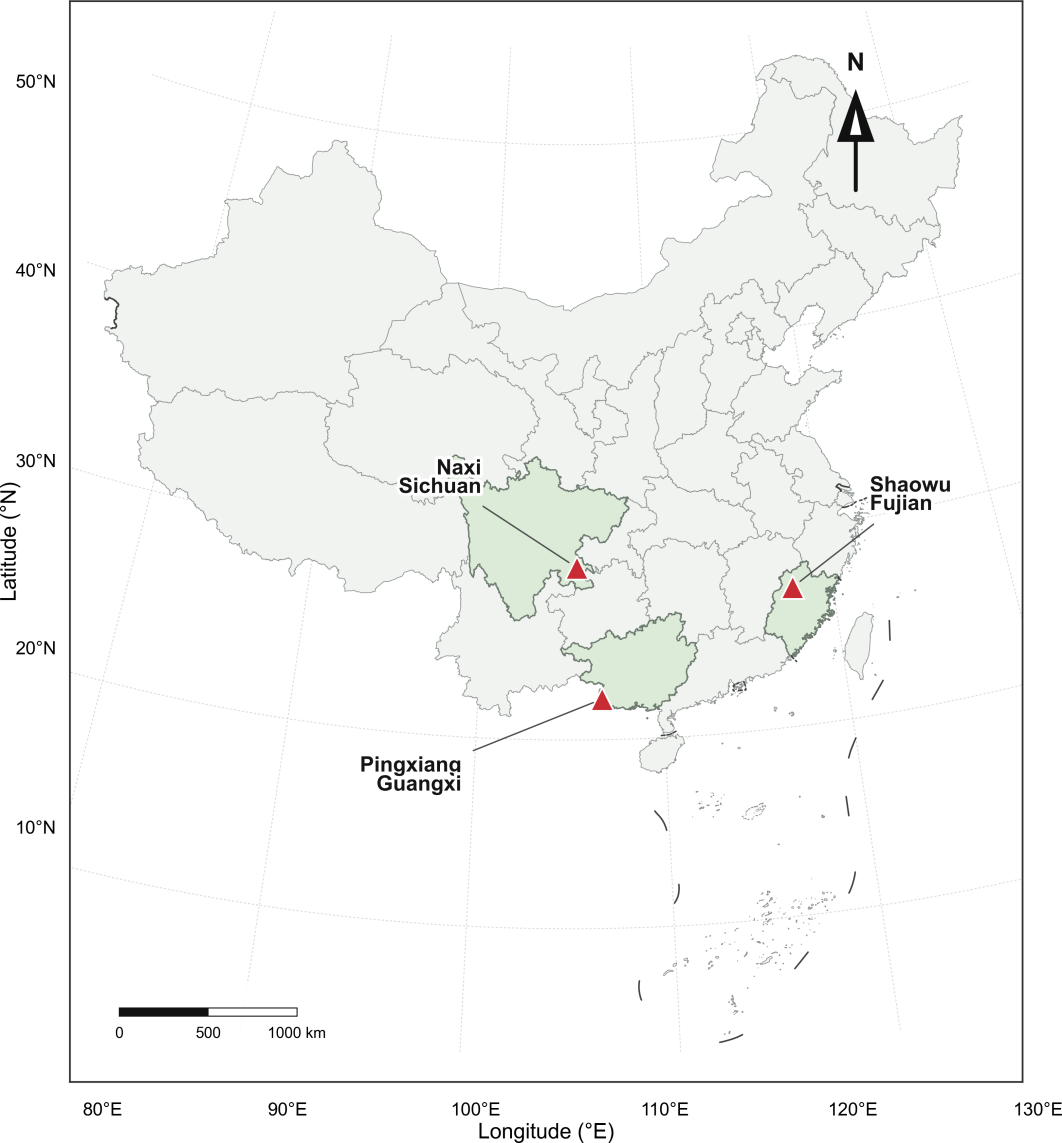
Geographic locations of three Cunninghamia lanceolata plantation study sites in subtropical China. Naxi (Sichuan), Pingxiang (Guangxi), and Shaowu (Fujian) are marked by red triangles, with the corresponding provinces shaded light green. The map uses an Albers equal-area conic projection. Boundary data: National Platform for Common Geospatial Information Services (Tianditu; GS(2024)0650); boundary geometry was not manually altered.

The study was conducted in three subtropical monsoon regions: Pingxiang in Guangxi, Shaowu in Fujian, and Naxi in Sichuan, representing the southern, central-eastern, and central parts of the Chinese fir distribution range (**Figure 1**). The main characteristics of each site are summarized in **Table 1**. Long-term plots were established in spring 1981 using one-year-old Chinese fir seedlings. At each site, 15 plots were established in a randomized complete block design with three blocks, with each block containing one replicate of all five initial planting densities (i.e., three replicate plots per density per site). The five planting densities were 1,667, 3,333, 5,000, 6,667, and 10,000 trees ha^-1^. Each plot measured 600 m² (20 × 30 m) and was surrounded by two buffer rows of similarly treated trees. Height (H) and diameter at breast height (DBH, 1.3 m) were recorded for all standing trees taller than 3 m.

**Table 1:** Overview of site conditions and stand characteristics of Chinese fir plantations.

Region	Latitude (°N)	Longitude (°E)	Main landform	Average elevation (m)	MAT (°C)	MAP (mm)	Mean site index (m)	Age (years)	Mean DBH (cm)	Mean height (m)	Initial planting density (trees ha^-1^)
Sichuan	28.78	105.38	Low mountain	440	18.48	1147	12.71	38	14.25	13.12	1,667, 3,333, 5,000, 6,667, 10,000
Guangxi	22.10	106.72	Low mountain	460	21.82	1313	15.11	35	17.23	17.81	1,667, 3,333, 5,000, 6,667, 10,000
Fujian	27.08	117.72	Low mountain	300	18.88	1831	17.74	41	23.87	22.84	1,667, 3,333, 5,000, 6,667, 10,000

DBH, diameter at breast height; MAT, mean annual temperature; MAP, mean annual precipitation.

### 2.2 Field data collection

#### 2.2.1 Classification of tree dominance classes

Tree dominance classes were determined by relative diameter *d_rel i_* = *d_i_/D*, where *d_i_* is the DBH of tree *i* and *D* represents the stand mean DBH (Ding et al., 1980). Trees were classified into four dominance classes based on their *d_rel i_* values: dominant (D, *d_rel i_* ≥ 1.336), co-dominant (CD, 1.026 ≤ *d_rel i_* < 1.336), intermediate (I, 0.712 ≤ *d_rel i_* < 1.026), and suppressed (S, *d_rel i_* < 0.712) (Ding et al., 1980). For each dominance class within each plot, we calculated mean DBH and height to estimate biomass, carbon storage, and allocation fractions.

#### 2.2.2 Estimation of biomass allocation fractions and carbon allocation fractions

The biomass of leaves (B_L_), branches (B_Br_), stems (B_S_), roots (B_R_), and total biomass (B_T_), as well as the carbon storage of leaves (C_L_), branches (C_Br_), stems (C_S_), roots (C_R_), and total carbon storage (C_T_) were estimated using region-specific additive biomass and carbon storage equations previously developed for mature Chinese fir plantations in different production regions of China (Lv and Duan, 2024). These equations were calibrated from destructive samples collected in the buffer rows of the same long-term density experiments used in the present study, and the buffer rows shared the same planting design as the measured plots. The equations were applied to DBH and height data from 4,029 living trees to derive organ-specific biomass, carbon stocks, and allocation fractions.

Biomass allocation fractions (BAFs) were calculated as the ratio of organ biomass to total tree biomass (B_T_): leaf biomass fraction (LMF = B_L_/B_T_), branch biomass fraction (BMF = B_Br_/B_T_), stem biomass fraction (SMF = B_S_/B_T_), and root biomass fraction (RMF = B_R_/B_T_). The biomass root-to-shoot ratio was calculated as R/S = B_R_/(B_S_+B_L_+B_Br_). Similarly, carbon allocation fractions (CAFs) were calculated as the ratio of organ carbon to total tree carbon, including leaf carbon fraction (LCF = C_L_/C_T_), branch carbon fraction (BCF = C_Br_/C_T_), stem carbon fraction (SCF = C_S_/C_T_), and root carbon fraction (RCF = C_R_/C_T_), with carbon root-to-shoot ratio calculated as C-R/S = C_R_/(C_S_+C_L_+C_Br_). For clarity, we compiled a list of abbreviations and definitions for stand structure and biomass/carbon allocation variables in **Supplementary Table S1**.

The additive equations applied in this study were parameterized at the regional scale rather than independently for each combination of planting density, site quality, and climate. Consequently, the present analysis does not test whether the allometric relationships differ among the specific treatment combinations. Instead, it evaluates how allometry-derived allocation fractions vary across structural and environmental gradients, including planting density, dominance class, site index, and climate. Therefore, the results should be interpreted as patterns in allometry-derived allocation indicators rather than as direct treatment-specific physiological responses.

### 2.3 Climate and stand drivers

Climatic variables for each sampling site were obtained using the ClimateAP platform (http://climateap.net/) (Wang et al., 2012). This tool provides spatially interpolated historical and projected climate data across the Asia Pacific region based on geographical coordinates specific to each site, including longitude, latitude, and elevation. To characterize climatic gradients associated with variation in the allocation fractions of *Cunninghamia lanceolata* plantations, we extracted annual, seasonal, and monthly climate records from ClimateAP. Key climatic predictors included mean annual temperature (MAT), mean annual precipitation (MAP), degree days above 5 °C (DD5), maximum temperature in July (Tmax_07), summer maximum temperature (Tmax_sm), spring precipitation (PPT_sp), and climatic moisture deficit (CMD). The climatic variables used to assess the effects of climate on biomass and carbon allocation are listed in **Supplementary Table S2**.

Site index (SI) was used as a measure of site quality because it is commonly defined from dominant stand height at a reference age (Kayahara et al., 1998; Li et al., 2023). In this study, SI was defined as the dominant height at a reference age of 20 years. Dominant height was calculated as the mean height of the six tallest individuals in each plot, and this metric at age 20 was used as the site index.

The stand density index (SDI) quantifies the number of trees per unit area when the quadratic mean diameter of a stand is standardized to a reference value (Woodall et al., 2003). This index was first proposed by Reineke (1933) based on empirical studies of even-aged, fully stocked pure stands across multiple species, and is expressed through a log-linear relationship between the number of trees per hectare (N) and the quadratic mean diameter (D_g_), expressed as: *ln (N) = -β ln (D_g_)* + *k*. SDI was computed as follows:


$$SDI=N{\left(\frac{D_{g}}{D_{0}}\right)}^{\beta }$$

where *N* is the number of trees per hectare; *D_g_* is the quadratic mean diameter at breast height (cm); *D_0_* is the reference diameter, which is typically set to 20 cm for Chinese fir; β is the self-thinning rate of the stand.

### 2.4 Statistical analysis

#### 2.4.1 Organ-specific allocation across dominance classes

**Figure 2:**
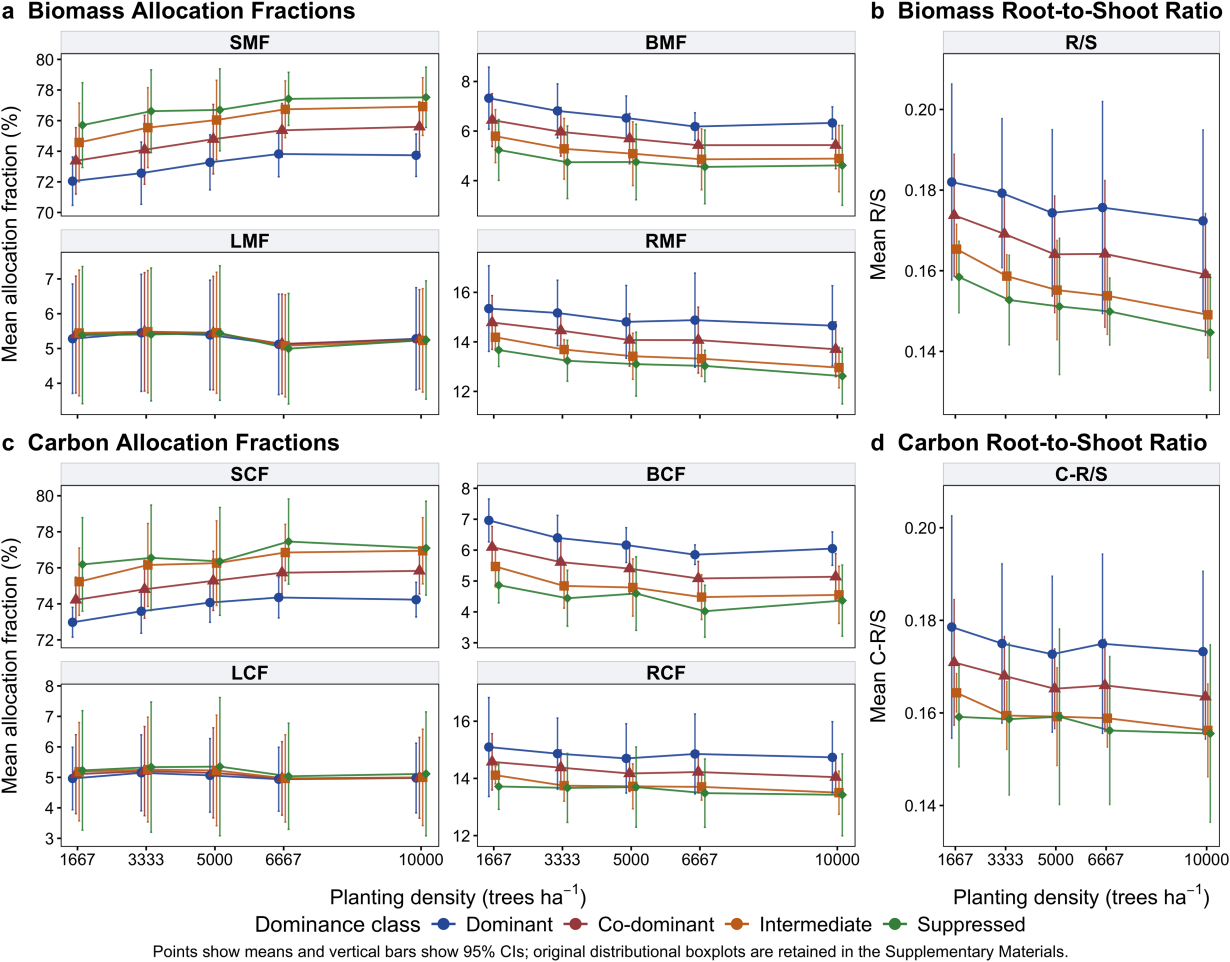
Density-related variation in biomass and carbon allocation fractions across dominance classes. Mean allocation fractions of Chinese fir trees across the initial planting-density gradient and four dominance classes. Panels show: **(a)** biomass allocation fractions, **(b)** biomass root:shoot ratio, **(c)** carbon allocation fractions, and **(d)** carbon root:shoot ratio. Points represent dominance-class-specific means, and vertical bars indicate 95% confidence intervals. Planting density is expressed in trees ha^-1^. SMF, stem biomass fraction; BMF, branch biomass fraction; LMF, leaf biomass fraction; RMF, root biomass fraction; SCF, stem carbon fraction; BCF, branch carbon fraction; LCF, leaf carbon fraction; RCF, root carbon fraction; R/S, biomass root:shoot ratio; C-R/S, carbon root:shoot ratio.

To compare BAFs and CAFs across dominance classes in mature plantations, we used a one-way analysis of variance (ANOVA) followed by Duncan’s multiple range test (P < 0.05; Duncan, 1955). All statistical analyses were conducted using SPSS 22.0 (IBM Corp., Armonk, NY, USA). To summarize variation in allocation fractions associated with initial planting density and dominance class, we plotted the mean values for each dominance class along the initial planting density gradient, with 95% confidence intervals shown as vertical bars (**Figure 2**). For the mean trends presented in **Figure 2**, we further tested the effects of planting density, dominance class, and their interaction for each allocation indicator. The corresponding ANOVA P values are provided in **Supplementary Table S3b**.

#### 2.4.2 Interactive effects of density and site index

**Figure 3:**
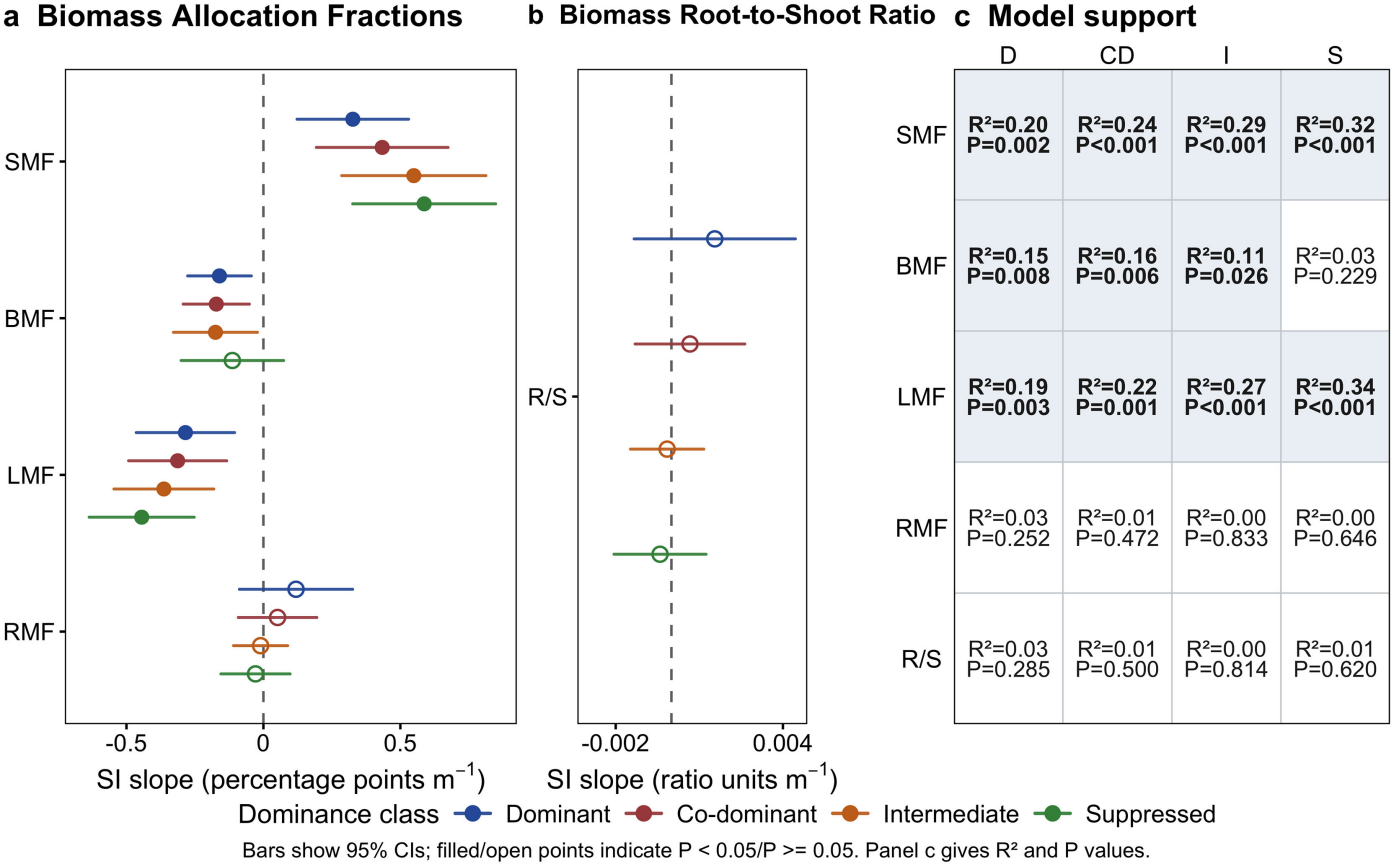
Site-index effects on biomass allocation fractions across dominance classes. Dominance-class-specific slopes from linear regressions between site index and biomass allocation indicators. Panel **(a)** shows slope estimates for biomass allocation fractions, panel **(b)** shows slope estimates for the biomass root:shoot ratio, and panel **(c)** reports the corresponding R² and P values for each regression. Points indicate slope estimates per 1 m increase in site index, and horizontal bars indicate 95% confidence intervals. The vertical dashed line represents a zero slope. Filled points indicate significant regressions (P < 0.05), whereas open points indicate non-significant regressions (P ≥ 0.05). SI, site index; SMF, stem biomass fraction; BMF, branch biomass fraction; LMF, leaf biomass fraction; RMF, root biomass fraction; R/S, biomass root:shoot ratio; D, dominant; CD, co-dominant; I, intermediate; S, suppressed.

**Figure 4:**
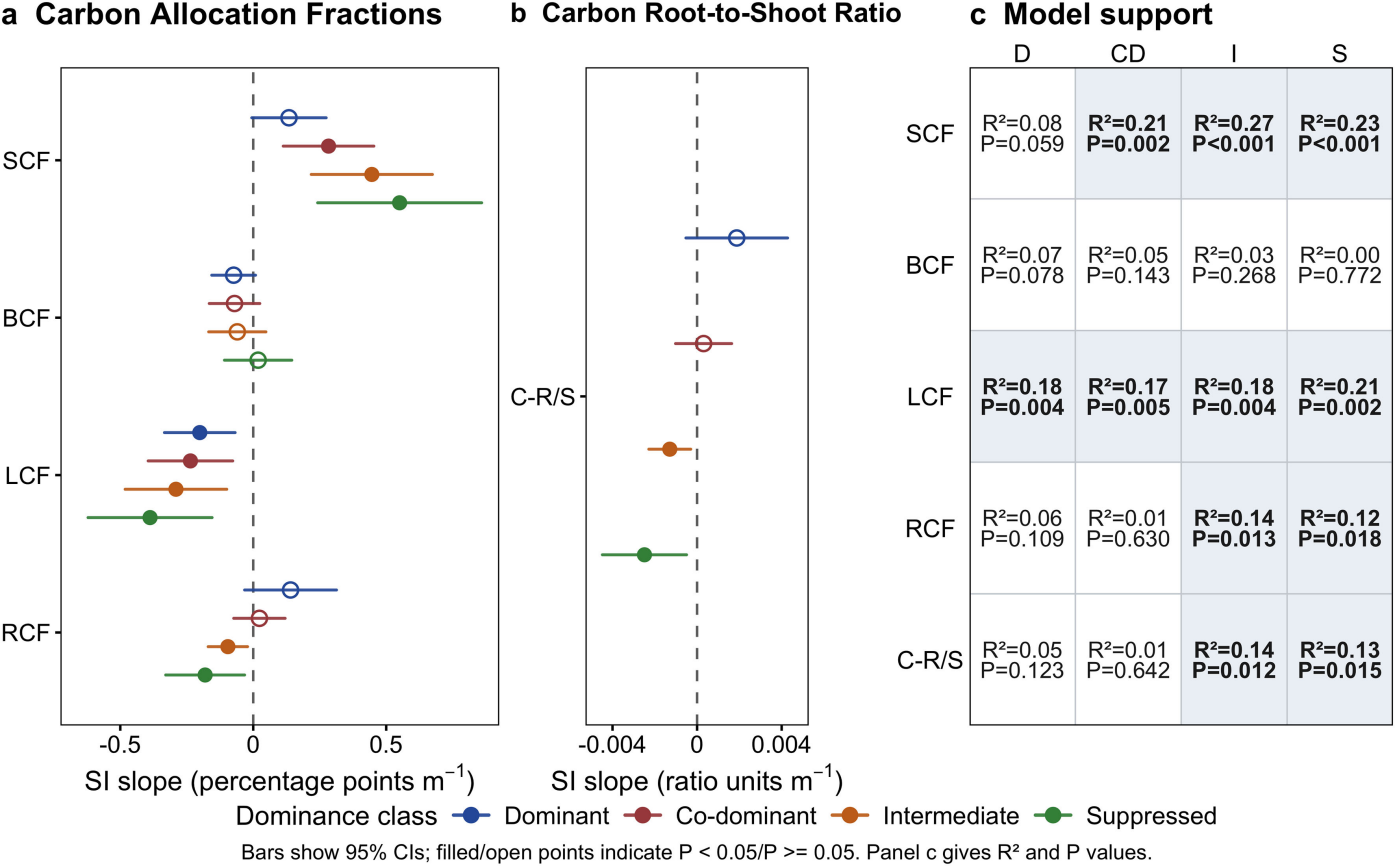
Site-index effects on carbon allocation fractions across dominance classes. Dominance-class-specific slopes from linear regressions between site index and carbon allocation indicators. Panel **(a)** shows slope estimates for carbon allocation fractions, panel **(b)** shows slope estimates for the carbon root:shoot ratio, and panel **(c)** reports the corresponding R² and P values for each regression. Points indicate slope estimates per 1 m increase in site index, and horizontal bars indicate 95% confidence intervals. The vertical dashed line represents a zero slope. Filled points indicate significant regressions (P < 0.05), whereas open points indicate non-significant regressions (P ≥ 0.05). SI, site index; SCF, stem carbon fraction; BCF, branch carbon fraction; LCF, leaf carbon fraction; RCF, root carbon fraction; C-R/S, carbon root:shoot ratio; D, dominant; CD, co-dominant; I, intermediate; S, suppressed.

Dirichlet regression models implemented in the R package “DirichletReg” were used only to generate bivariate response surfaces for visualizing the joint patterns of allocation fractions across planting density and site index (**Supplementary Figures S1**, **S2**). These models were not used as the primary inferential framework. To quantify site-index-related trends in allocation fractions, we fitted separate linear regressions for each dominance class and allocation variable. Regression slopes and 95% CIs were displayed in **Figures 3** and **4**, and the corresponding coefficients of determination (R^2^) and P values were reported in the regression-support panels. Full regression statistics are provided in **Supplementary Table S5**. All plots were generated using the “ggplot2” package in R version 4.4.1.

#### 2.4.3 Drivers associated with estimated biomass and carbon allocation fractions

**Figure 5:**
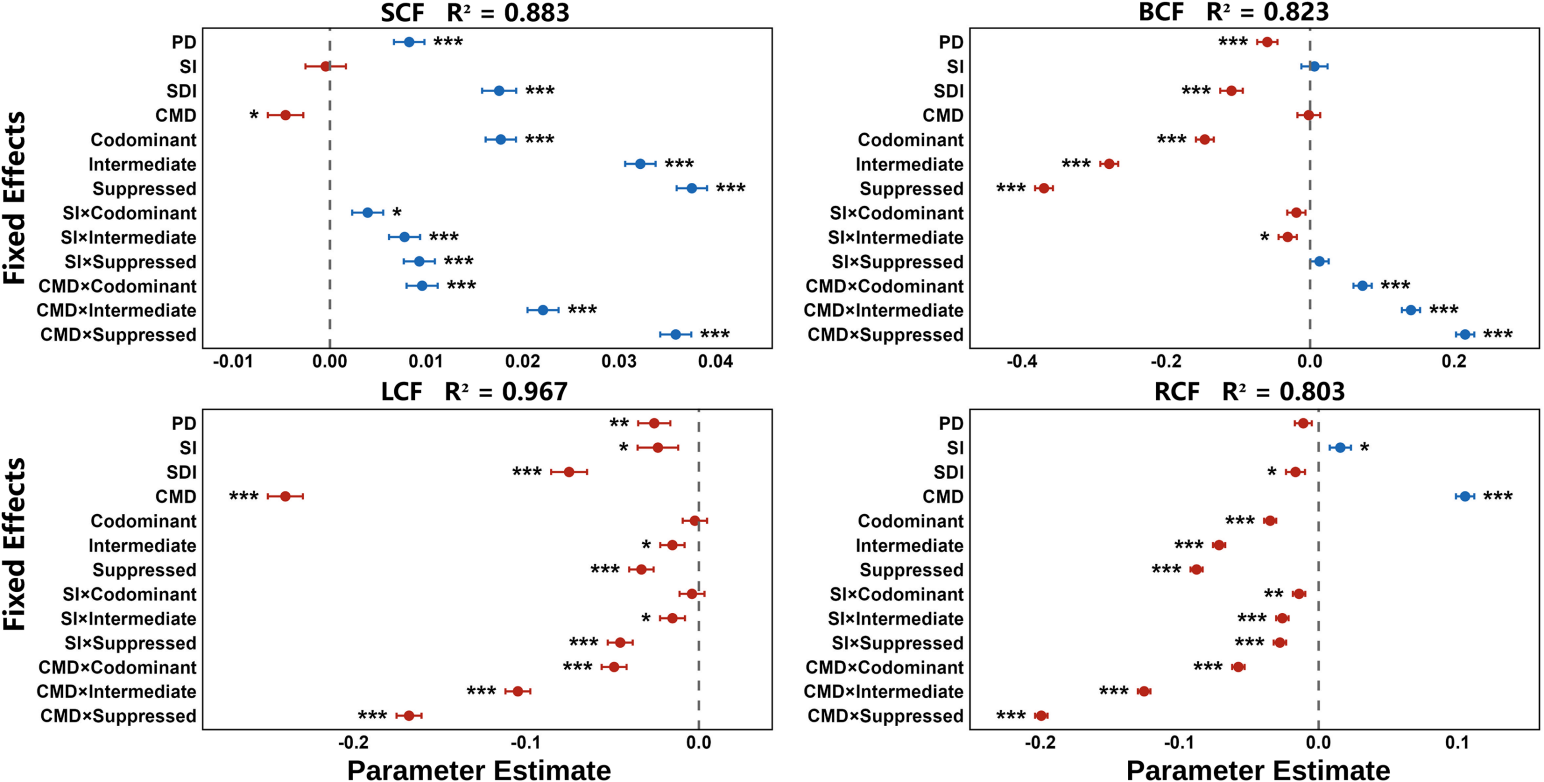
Estimated fixed effects and 95% confidence intervals for the linear mixed-effects models predicting CAFs. Positive effects are shown in blue and negative effects in red. Asterisks indicate statistical significance: *significance at P < 0.05; **significance at P < 0.01; ***significance at P < 0.001. R^2^ values represent the marginal R^2^ (variance explained by fixed effects). PD, initial planting density; SI, site index; SDI, stand density index; SCF, stem carbon fraction; BCF, branch carbon fraction; LCF, leaf carbon fraction; RCF, root carbon fraction.

**Figure 6:**
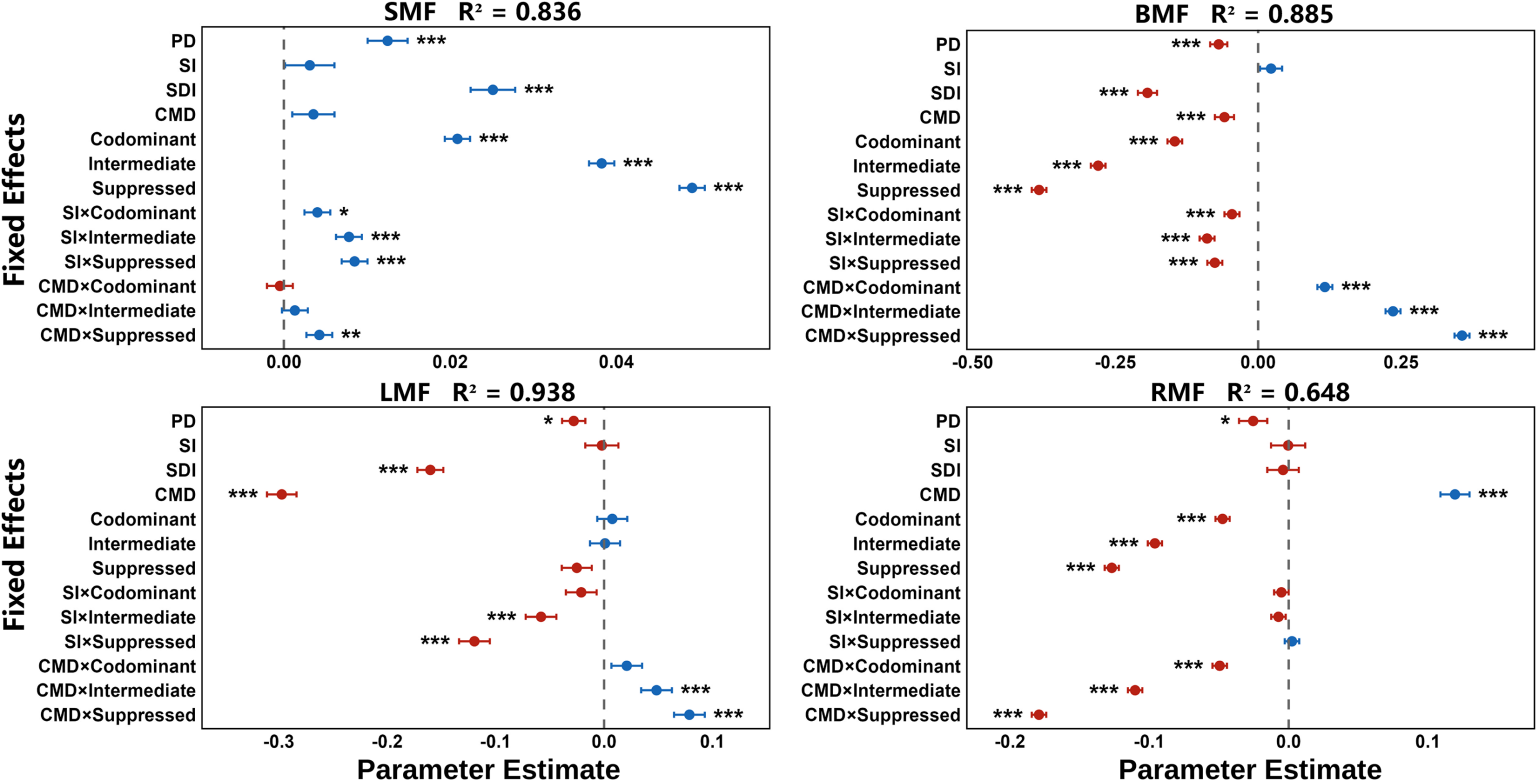
Estimated fixed effects and 95% confidence intervals for the linear mixed-effects models predicting BAFs. Positive effects are shown in blue and negative effects in red. Asterisks indicate statistical significance: *significance at P < 0.05; **significance at P < 0.01; ***significance at P < 0.001. R^2^ values represent the marginal R^2^ (variance explained by fixed effects). PD, initial planting density; SI, site index; SDI, stand density index; SMF, stem biomass fraction; BMF, branch biomass fraction; LMF, leaf biomass fraction; RMF, root biomass fraction.

To evaluate the main and interactive associations of dominance class, planting density, site quality, and climate with organ-specific allocation fractions, we fitted linear mixed-effects models (LMEs) using the R package ‘lme4’ (**Figures 5**, **6**). Our objective was to evaluate the relative associations of stand structure, site index, climate, and dominance class with allocation fractions, rather than to build a purely predictive model.

Prior to model fitting, we addressed potential multicollinearity among the climate predictors. We first removed highly correlated climate variables using pairwise Pearson correlations (|r| > 0.70), and then screened the remaining candidates together with other fixed effects using variance inflation factors (VIF), excluding predictors with VIF > 5. Climatic moisture deficit (CMD) was defined as the cumulative monthly excess of reference evaporative demand over precipitation and represents the additional moisture required for vegetation growth beyond rainfall. Annual CMD was retained in the final LME as an interpretable index of climatic water limitation. To address the possibility that annual CMD could obscure seasonal moisture limitation, we also performed sensitivity analyses in which annual CMD was replaced by seasonal CMD variables and by individual annual or seasonal climate variables. The unstandardized annual and seasonal ClimateAP variables used in these sensitivity analyses are listed in **Supplementary Table S4**, and the model-comparison and slope results are summarized in **Supplementary Tables S6**, **S7**. These climate-sensitivity analyses were used to evaluate whether the broad direction of CMD-associated allocation patterns was robust to alternative climate-variable specifications, rather than to identify independently replicated climatic drivers.

Before the statistical analyses, the response variables were natural-log-transformed to improve linearity and normality, and all continuous predictors were Z-score standardized (mean = 0, SD = 1) for numerical stability and comparability of effect sizes. Dominance class, PD, SI, CMD, and SDI were included as fixed effects, and plot was included as a random intercept. Although dominance class is an ordered variable, its levels are not equally spaced in biomass or size; therefore, we modeled it as a categorical factor with “dominant” as the reference. With this parameterization, the intercept represents the dominant class, and the coefficients for the remaining classes quantify their deviations relative to dominant trees, avoiding artificial linear spacing across classes and yielding class-specific estimates (Jazbec et al., 2023). Because climate, stand age, elevation, latitude, longitude, and site-level mean tree-size variables were defined at the site level, their coefficients were identified from only three regional values and should be interpreted as site-level associations rather than independently replicated causal effects. These variables also partly covaried with climate and site quality, increasing the risk of over-parameterization. Mean DBH and height posed an additional issue because they were direct inputs to the allometric equations used to estimate organ biomass and carbon storage; including them again in allocation-fraction models could therefore introduce circular adjustment. We excluded these variables from the final inferential models and used one-at-a-time sensitivity analyses to assess whether their inclusion changed the main CMD-associated directions (**Supplementary Tables S8**, **S9**). Because several sensitivity models included site-level predictors with only three regional values and produced singular-fit warnings, these analyses were interpreted as directional robustness checks rather than independent inference for individual site-level predictors.

Model goodness-of-fit was evaluated using the marginal coefficient of determination (R^2^), which quantifies the proportion of variance explained solely by the fixed effects (Nakagawa and Schielzeth, 2013), ensuring the reported R^2^ values reflect the explanatory power of our focal drivers without including random plot variation.

## 3 Results

### 3.1 Organ-specific biomass and carbon allocation across dominance classes

**Table 2** summarizes organ-specific biomass and carbon allocation fractions for Chinese fir trees across dominance classes. Across all sampled sites, SMF and SCF increased steadily from dominant to suppressed trees. Conversely, BMF, BCF, LCF, RCF, and the R/S ratio decreased from dominant to suppressed trees (**Table 2**). In the pooled dataset, SCF increased and RCF decreased from dominant to suppressed trees. However, the Sichuan site showed the opposite pattern: SCF was highest in dominant trees and declined toward suppressed trees, whereas RCF increased from dominant to suppressed trees. At the site level, Sichuan also differed in the direction of the R/S dominance-class trend: R/S increased from dominant to suppressed trees (0.1484 ± 0.0096 to 0.1624 ± 0.0123), whereas it decreased across the same dominance gradient in Fujian (0.1725 ± 0.0052 to 0.1534 ± 0.0064) and Guangxi (0.2093 ± 0.0195 to 0.1383 ± 0.0172; **Supplementary Table S3**).

**Table 2:** BAFs and CAFs of Chinese fir across dominance classes.

Dominance class	Stem		Branch		Leaf		Root		R/S	
	SMF (%)	SCF (%)	BMF (%)	BCF (%)	LMF (%)	LCF (%)	RMF (%)	RCF (%)	R/S	C-R/S
Dominant	73.09 ± 2.19c	73.84 ± 1.41c	6.64 ± 1.22a	6.29 ± 0.83a	5.30 ± 1.93a	5.02 ± 1.41a	14.97 ± 2.03a	14.85 ± 1.72a	0.1767 ± 0.0283a	0.1749 ± 0.0239a
Co-dominant	74.64 ± 2.65b	75.17 ± 1.85b	5.79 ± 1.27b	5.47 ± 0.94b	5.34 ± 1.96a	5.08 ± 1.69a	14.22 ± 1.39b	14.28 ± 0.94b	0.1660 ± 0.0191b	0.1667 ± 0.0128b
Intermediate	75.96 ± 3.02a	76.29 ± 2.57a	5.18 ± 1.57bc	4.83 ± 1.05c	5.34 ± 2.06a	5.13 ± 2.04a	13.52 ± 0.96c	13.76 ± 0.77bc	0.1564 ± 0.0127c	0.1596 ± 0.0103bc
Suppressed	76.79 ± 3.06a	76.73 ± 3.40a	4.78 ± 1.84c	4.46 ± 1.22c	5.29 ± 2.27a	5.21 ± 2.53a	13.13 ± 1.22c	13.60 ± 1.53c	0.1514 ± 0.0160c	0.1577 ± 0.0206c

SMF, stem biomass fraction; BMF, branch biomass fraction; LMF, leaf biomass fraction; RMF, root biomass fraction; SCF, stem carbon fraction; BCF, branch carbon fraction; LCF, leaf carbon fraction; RCF, root carbon fraction; R/S, root-to-shoot ratio of biomass; C-R/S, root-to-shoot ratio of carbon. Data are presented as average ± standard deviation. Different letters within each column indicate significant differences (P < 0.05).

### 3.2 Density and dominance class related variation in organ-level allocation fractions

Biomass and carbon allocation fractions differed among dominance classes across the planting-density gradient (**Figure 2**; **Supplementary Table S3b**), with the underlying distributional patterns shown in **Supplementary Figure S3**. Across density levels, SMF and SCF were generally higher in intermediate and suppressed trees than in dominant trees, whereas BMF, BCF, RMF, RCF, R/S, and C-R/S were generally higher in dominant trees. Two-way ANOVA showed significant dominance-class effects for most stem, branch, root, and root:shoot indicators (SMF, BMF, RMF, R/S, SCF, BCF, RCF, and C-R/S), whereas LMF and LCF showed no significant dominance-class effect (**Supplementary Table S3b**). Planting density had a significant main effect on SMF (P < 0.05) and BCF (P < 0.01), with weak or non-significant effects on the other indicators. The density × dominance-class interaction was not significant for any indicator, indicating broadly similar density-related trends across dominance classes. With increasing planting density, SMF and SCF increased before levelling off at medium-to-high densities, whereas branch, root, and root-to-shoot allocation declined; LMF and LCF varied only weakly across the gradient.

### 3.3 Site index related variation in allocation fractions across dominance classes

Dominance-class-specific regressions related site index (SI) to several biomass and carbon allocation fractions (**Figures 3**, **4**; **Supplementary Table S5**). For biomass allocation, SMF increased with SI in all dominance classes (R^2^ = 0.20–0.32, P < 0.01) and LMF decreased across all classes (R^2^ = 0.19–0.34, P < 0.01). BMF also decreased with SI in dominant, co-dominant, and intermediate trees (R^2^ = 0.11–0.16, P < 0.05) but not in suppressed trees, whereas RMF and R/S were not significantly related to SI. For carbon allocation, SCF increased with SI in co-dominant, intermediate, and suppressed trees (R^2^ = 0.21–0.27, P < 0.01), but only marginally and non-significantly in dominant trees. LCF decreased in all dominance classes (R^2^ = 0.17–0.21, P < 0.01), whereas BCF showed no significant relationship with SI. RCF and C-R/S declined only in intermediate and suppressed trees (P < 0.05), not in dominant or co-dominant trees.

### 3.4 Mixed effects model results for estimated organ-level allocation

Parameter estimates for the fixed effects in the linear mixed-effects models are presented in **Figures 5** and **6**. The mixed effects models identified consistent associations of allocation fractions with PD, SI, SDI, CMD, and dominance class. Variance inflation factors for the final predictors were low (VIF < 3), suggesting that multicollinearity among the retained predictors did not substantially affect model interpretation. The intensity of competition (PD and SDI) was significantly associated with higher stem biomass and carbon allocation and lower leaf, branch, and root allocation fractions (P < 0.05). As dominance decreased from dominant to suppressed trees, SMF and SCF increased significantly, whereas the biomass and carbon fractions allocated to branches, leaves, and roots decreased significantly. Allocation responses to CMD and SI differed among dominance classes. Under increasing CMD, dominant trees showed weak changes in branch allocation and relatively stable or slightly positive associations with root allocation, whereas non-dominant trees showed higher branch fractions and lower estimated root fractions. SCF also showed class-dependent divergence, declining in dominant trees but increasing in other dominance classes. Along the SI gradient, higher SI generally favored stem allocation over leaf allocation across dominance classes, with the strongest shifts occurring in suppressed trees. Branch and root allocation showed contrasting dominance-specific slopes, changing from positive associations in dominant trees to negative responses in intermediate and suppressed trees.

Sensitivity analyses using seasonal CMD and alternative climate variables supported the broad direction of most allocation responses but showed that seasonal effects were not identical across all variables (**Supplementary Tables S6**, **S7**). In suppressed trees, annual CMD was negatively associated with RCF and RMF but positively associated with SCF and SMF, and autumn or winter CMD provided the closest seasonal support for several of these responses. One-at-a-time addition of stand age, elevation, latitude, longitude, mean DBH, mean height, DBH, and height preserved the annual CMD direction for all eight checks in BMF, LMF, RMF, R/S, LCF, RCF, and C-R/S, with limited exceptions for SMF, SCF, and BCF (**Supplementary Tables S8**, **S9**). The mixed-effects models indicate that stem allocation increased with stronger competition and higher site quality, whereas belowground allocation responses were most pronounced in intermediate and suppressed trees under site-index and CMD gradients.

## 4 Discussion

Drawing on multi-regional data from mature Chinese fir plantations, this study examined how initial planting density (PD), site index (SI), climatic moisture deficit (CMD), and dominance class were associated with variation in organ-level biomass and carbon allocation. Across these gradients, stronger competition and higher site index were generally associated with greater stem allocation and lower leaf and root allocation, and these shifts tended to become more pronounced from dominant to suppressed trees. These overall tendencies differed among dominance classes, with suppressed trees showing stronger variation in allocation fractions along environmental gradients than dominant trees. Dominant trees showed relatively stable root fractions across the CMD gradient, whereas suppressed trees showed stronger shifts toward higher stem fractions and lower belowground allocation under higher moisture deficit. Because a disproportionate share of stand-level carbon stock is stored in the largest individuals (Fedrigo et al., 2014), the relative stability of allocation in dominant trees may help buffer plantation carbon stocks, whereas subordinate cohorts may represent a more climate-sensitive component. These interpretations, however, should be understood within the limits of locally estimated allocation indicators derived from region-specific allometric equations rather than direct destructive measurements for all treatment combinations.

### 4.1 Variation in allocation fractions along density, site quality, and climate gradients

Increasing planting density (PD) was associated with higher stem allocation fractions and lower branch and root allocation fractions. This trend aligns with findings reported by Wang et al. (2022) and may reflect a shade-avoidance tendency under intensified light competition, whereby trees maintain vertical growth at the expense of crown expansion and root development (Dybzinski et al., 2015; Wertz et al., 2020). Consistent with evidence that crowding shifts allometry toward height growth (Trouvé et al., 2015), the observed increase in stem fractions under higher PD may indicate a structural adjustment to secure canopy position at the expense of resource acquisition organs.

The results provide only partial support for optimal partitioning theory (OPT) and functional equilibrium frameworks, which predict greater relative investment in organs responsible for acquiring the most limiting resource (Bloom et al., 1985; Chapin et al., 1987; Poorter et al., 2012). The SI-related trends were broadly consistent with this expectation: higher site index was associated with greater stem allocation and lower root allocation, suggesting that on more productive sites rapid canopy development and light competition may favor investment in vertical structure, whereas poorer sites may maintain greater belowground allocation for resource capture (Fang et al., 2018; Wang et al., 2022). The CMD-related patterns were less consistent. Dominant trees showed relatively stable, or slightly increasing, root biomass and carbon fractions as CMD increased, compatible with limited belowground adjustment under water limitation. Suppressed trees, in contrast, showed lower belowground fractions under higher CMD, departing from the OPT prediction of increased root allocation. This suggests that allocation in subordinate trees reflects the combined effects of water availability, size hierarchy, shade competition, and structural allometry. Under concurrent light and water limitation, dominance status may modify or override simple resource-based expectations.

### 4.2 Dominance-class structure and allocation asymmetry

Even in even-aged monoculture plantations, asymmetric competition for light, water, and nutrients can stratify trees into dominance classes and generate persistent structural heterogeneity (Reynolds and Ford, 2005; Schwinning and Weiner, 1998; Trouvé et al., 2017; Umaña et al., 2021). Such dominance-related heterogeneity is directly relevant to allocation because tree social status influences crown development and access to belowground resources, along with size-related allometric trajectories (Naidu et al., 1998; Gargaglione et al., 2010; Trouvé et al., 2014; Deng et al., 2023; Lv et al., 2024). Dominance class therefore provides an important structural context for interpreting organ-level allocation patterns in mature Chinese fir plantations.

Across density levels, dominance-class differences remained evident even where density-related changes in allocation tended to level off at medium-to-high planting densities. Our analysis indicates that stem fractions increased stepwise from dominant to suppressed trees, while branch, leaf, and root fractions decreased (Naidu et al., 1998; Vanninen and Makela, 2000; Wertz et al., 2020). This pattern is consistent with stronger aboveground structural emphasis in subordinate trees, whereas dominant individuals, having secured light access, maintained relatively larger crown- and root-related fractions (Poorter and Nagel, 2000).

### 4.3 Site-specific and belowground responses under environmental stress

Mixed-effects models indicated that SI-related variation in allocation differed among dominance classes. The contrasting Sichuan pattern, in which dominant trees showed higher SCF whereas lower dominance classes showed higher RCF, is unlikely to be explained by stand age alone: Sichuan stands were 38 years old, compared with 35 years in Guangxi and 41 years in Fujian. Elevation also differed little between Sichuan and Guangxi (440 vs. 460 m). Instead, this local anomaly is more consistent with the combined effects of lower site quality, smaller tree size, dominance-related allometry, and region-specific biomass-carbon relationships. Site quality and soil resource availability influence biomass partitioning and root-shoot balance, particularly where nutrient or water availability constrains growth (Kayahara et al., 1998; Deng et al., 2023; Qi et al., 2019; Ni et al., 2022). Dominant and suppressed trees can also differ in biomass partitioning because asymmetric competition alters crown development, stem investment, and access to belowground resources (Naidu et al., 1998; Gargaglione et al., 2010; Trouvé et al., 2015). Sichuan had the lowest annual CMD among the study regions (145.92 mm; **Supplementary Table S4**), together with lower MAP and cooler growing-season conditions than Guangxi. The observed anomaly therefore cannot be attributed to CMD range alone. Because the allocation fractions in this study were derived from region-specific allometric carbon equations, part of the Sichuan pattern may also reflect regional tree-size structure and allometric-carbon relationships rather than a single physiological driver (Lv and Duan, 2024; Lv et al., 2024). We therefore interpret this pattern cautiously and recommend direct tests using soil, fine-root, and mycorrhizal measurements (Gong et al., 2023; Genre et al., 2020; Cheng et al., 2021).

Climate-related allocation patterns also differed among dominance classes. Dominant trees displayed relatively stable root fractions under moisture stress, whereas suppressed trees showed stronger declines in RMF and RCF accompanied by higher stem allocation. This pattern is contrary to the simplest OPT expectation for water limitation and suggests that under simultaneous light and water limitation, suppressed trees may prioritize aboveground competitive persistence over drought acclimation, potentially trading root-based hydraulic resilience for stem investment (Poorter and Nagel, 2000; Umaña et al., 2021). Such a shift may be associated with reduced access to soil moisture and greater vulnerability during prolonged dry periods (McDowell et al., 2018). Similar status-dependent climate sensitivity has also been reported in other forests, where suppressed individuals show greater drought-related growth limitation and mortality risk than dominant trees (Cavin et al., 2013; Lebourgeois et al., 2014). Allocation patterns in Chinese fir plantations are not solely associated with size-related allometry, but also vary systematically with competitive position within the stand hierarchy (Weiner, 2004), with climate and competition jointly contributing to greater allocation asymmetry (Carnwath and Nelson, 2016).

This class-dependent trade-off may have important implications for mortality dynamics and the stability of plantation carbon stocks. The combination of lower root fractions and higher stem fractions under high CMD suggests that suppressed trees may represent a relatively high-risk cohort under climatic drying. This interpretation is consistent with long-term observations showing that dominant trees often recover more rapidly after drought, whereas suppressed trees experience longer-lasting growth limitation or mortality (Cavin et al., 2013; Trouvé et al., 2025). Climatic drying may reshape competitive relationships within stands and influence carbon stock trajectories through interactions among stand structure, site productivity, and management history. Such interactions call for vulnerability assessments that explicitly integrate climatic drivers with competition processes (Rubio-Cuadrado et al., 2018; Socha et al., 2023; Vayreda et al., 2012).

Belowground carbon allocation may also be mediated by mycorrhizal fungi, a mechanism not directly measured in the present study. Mycorrhizal symbioses receive host-derived photosynthate and can improve nutrient uptake, water acquisition, soil aggregation, and drought tolerance (Genre et al., 2020; Cheng et al., 2021). Drought can reshape belowground allocation by altering root biomass fractions, carbon transfer to fungal partners, and the efficiency of resource uptake. In our allometry-based framework, RMF and RCF capture coarse-root and root-system carbon fractions estimated from tree dimensions, but they do not quantify fine-root turnover, mycorrhizal colonization, or carbon allocated to external hyphal networks. A suppressed tree with a lower estimated root fraction may still differ in mycorrhizal dependence or fungal carbon cost. Combining biomass allocation, fine-root traits, soil properties, and mycorrhizal measurements would test whether mycorrhizal mediation helps explain site-specific allocation patterns such as those observed in Sichuan.

### 4.4 Management implications

Although allocation fractions were allometry-derived, our findings indicate that planting density, site index, climate, and dominance class are jointly associated with organ-level carbon allocation in mature Chinese fir plantations. Silvicultural strategies should weigh short-term stand volume alongside the structural conditions associated with longer-term carbon persistence. Given that stem wood constitutes the largest and most persistent carbon pool (Pugh et al., 2020), density management that maintains relatively high stem fractions without strongly reducing belowground allocation may reduce suppression and sustain stem carbon accumulation (Reich et al., 2014).

On highly productive sites, stem allocation increased, but root and crown allocation tended to decline, and differences among dominance classes became more pronounced. Under such conditions, raising planting density above moderate levels within the tested range may yield only limited additional gains in stem carbon while increasing suppression and structural imbalance, particularly in weaker cohorts (Harris, 2007; Li et al., 2024; Trouvé et al., 2014; Wertz et al., 2020). For such sites, maintaining a moderate initial density and considering early- to mid-rotation thinning from below may therefore be beneficial once strong size differentiation emerges (Li et al., 2023; Reynolds and Ford, 2005). On low-productivity or water-limited sites, relatively low initial density (e.g., 1,667–3,333 trees ha^-1^) may help maintain higher root fractions and root:shoot ratios, which may support resource acquisition and drought tolerance (Farooq et al., 2019; Qi et al., 2019; Sohn et al., 2016; Sun et al., 2025), whereas very high density can intensify moisture stress and accelerate self-thinning (Carnwath and Nelson, 2016; Cavin et al., 2013; Ni et al., 2022).

In our study, suppressed and intermediate trees exhibited the steepest declines in root allocation under climatic moisture deficit, consistent with empirical evidence that trees of lower social status are often more sensitive to drought, particularly in denser stands (Rubio-Cuadrado et al., 2018; Trouvé et al., 2017). Thinning in overly dense stands can prioritize the removal of suppressed and low-vigor intermediate trees, which can improve soil water availability for dominant and co-dominant crop trees and reduce drought-related hydraulic risk during dry periods (Harris, 2007; Li et al., 2024; Naidu et al., 1998; Schwinning and Weiner, 1998; Zhang et al., 2018). Forest carbon accounting and growth models that ignore dominance-class heterogeneity may miss systematic variation in allocation-related indicators, such as stem carbon fractions and root:shoot ratios, when relying on uniform stand-level parameterization. Integrating these indicators into density and thinning decision rules would better identify class-dependent drought vulnerability while maintaining productivity targets and improving carbon-stock estimates under carbon-neutrality-oriented planning.

## 5 Conclusion

This study examined how initial planting density, site index, climatic moisture deficit, and dominance class relate to organ-level biomass and carbon allocation in mature *Cunninghamia lanceolata* plantations across three subtropical sites. Allocation differed consistently among dominance classes: from dominant to suppressed trees, the stem biomass fraction rose from 73.1% to 76.8% while branch and root fractions declined. Higher site index and stronger competition were associated with the same direction of change (greater stem, lower leaf and root allocation), and responses to climatic moisture deficit depended on dominance class: dominant trees held root fractions relatively stable, whereas suppressed trees shifted carbon toward stems and away from roots. Allocation patterns in Chinese fir were therefore more closely associated with competitive position than with any single environmental gradient, and only partly matched optimal-partitioning expectations once social status was taken into account. Resolving allocation by dominance class identifies suppressed cohorts as the more drought-sensitive component of the stand. For management, moderate initial densities on productive sites and thinning that relieves excessive suppression may help maintain stand conditions associated with more balanced organ-level allocation and greater carbon-stock resilience. Because these patterns derive from allometric estimates rather than directly measured fluxes, they should be confirmed with direct belowground and turnover data. Maintaining root allocation and preventing severe suppression should therefore be considered in density-management decisions for Chinese fir plantations under a drying climate.

## Data Availability

The raw data supporting the conclusions of this article will be made available by the authors, without undue reservation.
