# Supplementary material for: Carbon allocation across dominance classes varies with planting density, site quality, and climatic moisture deficit in Cunninghamia lanceolata plantations
Source: Front Plant Sci. 2026 Jul 15;17:1878250. doi: 10.3389/fpls.2026.1878250 (PMC13414936; doi:10.3389/fpls.2026.1878250)
Supplement: Supplementary file 1 [file SupplementaryFile1.docx]

**Supplementary Materials**

**Table S1. Abbreviations and definitions used in the manuscript.**

| **Acronym** | **Full term / variable description** | **Acronym** | **Full term / variable description** | **Acronym** | **Full term / variable description** |
| --- | --- | --- | --- | --- | --- |
| D | Dominant trees | CD | Co-dominant trees | I | Intermediate trees |
| S | Suppressed trees | BAFs | Biomass allocation fractions | CAFs | Carbon allocation fractions |
| PD | Initial planting density (trees ha^-1^) | SDI | Stand density index | SI | Site index |
| B_T_ | Biomass of total tree | B_R_ | Biomass of root | B_L_ | Biomass of leaf |
| B_Br_ | Biomass of branch | B_S_ | Biomass of stem | C_T_ | Carbon storage of total tree |
| C_R_ | Carbon storage of root | C_L_ | Carbon storage of leaf | C_Br_ | Carbon storage of branch |
| C_S_ | Carbon storage of stem | LMF | Leaf biomass fraction (%) | BMF | Branch biomass fraction (%) |
| SMF | Stem biomass fraction (%) | RMF | Root biomass fraction (%) | R/S | Root-to-shoot biomass ratio |
| LCF | Leaf carbon fraction (%) | BCF | Branch carbon fraction (%) | SCF | Stem carbon fraction (%) |
| RCF | Root carbon fraction (%) | C-R/S | Root-to-shoot carbon ratio | CMD | Climatic moisture deficit |

**Table S2. Geographic information and selected site-level ClimateAP variables for the three study regions.**

| **Regions** | **Elevation** | **Latitude (°N)** | **Longitude (°E)** | **MAT (℃)** | **MAP (mm)** | **DD5 (℃)** | **Tmax_sm (℃)** | **PPT_sp (mm)** | **Tmax_07 (℃)** | **CMD (mm)** |
| --- | --- | --- | --- | --- | --- | --- | --- | --- | --- | --- |
| Fujian | 300 | 27.08 | 117.72 | 18.88 | 1831.15 | 5042.08 | 31.93 | 726.73 | 33.28 | 217.40 |
| Guangxi | 460 | 22.10 | 106.72 | 21.82 | 1312.65 | 6072.21 | 31.41 | 288.88 | 31.50 | 313.76 |
| Sichuan | 440 | 28.78 | 105.38 | 18.48 | 1147.38 | 4899.05 | 30.78 | 251.97 | 31.63 | 145.92 |

*MAT, mean annual temperature; MAP, mean annual precipitation; DD5, degree-days above 5 ℃; Tmax_sm, summer maximum temperature; PPT_sp, spring precipitation; Tmax_07, July maximum temperature; CMD, annual climatic moisture deficit.*

**Table S3. Biomass and carbon allocation fractions and root:shoot ratios across dominance classes in the three study regions.**

| **Region** | **Dominance class** | **Stem** | | **Branch** | | **Leaf** | | **Root** | | **R/S** | |
| --- | --- | --- | --- | --- | --- | --- | --- | --- | --- | --- | --- |
|  |  | SMF (%) | SCF (%) | BMF (%) | BCF (%) | LMF (%) | LCF (%) | RMF (%) | RCF (%) | R/S | C-R/S |
| Fujian | Dominant | 75.34 ± 1.03d | 75.18 ± 0.88c | 5.55 ± 0.62a | 5.55 ± 0.51a | 4.40 ± 0.17a | 4.49 ± 0.08a | 14.71 ± 0.38a | 14.78 ± 0.38a | 0.1725 ± 0.0052a | 0.1734 ± 0.0053a |
| Fujian | Co-dominant | 77.63 ± 1.10c | 77.11 ± 0.95b | 4.26 ± 0.59b | 4.52 ± 0.49b | 4.23 ± 0.16b | 4.46 ± 0.07a | 13.88 ± 0.44b | 13.91 ± 0.44b | 0.1613 ± 0.0059b | 0.1616 ± 0.0060b |
| Fujian | Intermediate | 79.47 ± 0.94b | 78.59 ± 0.82a | 3.39 ± 0.45c | 3.86 ± 0.39c | 3.86 ± 0.19c | 4.31 ± 0.09b | 13.29 ± 0.40c | 13.24 ± 0.40c | 0.1532 ± 0.0053c | 0.1526 ± 0.0054c |
| Fujian | Suppressed | 80.27 ± 0.98a | 79.03 ± 0.90a | 3.13 ± 0.44c | 3.83 ± 0.44c | 3.31 ± 0.19d | 4.02 ± 0.10c | 13.30 ± 0.48c | 13.12 ± 0.46c | 0.1534 ± 0.0064c | 0.1510 ± 0.0061c |
| Guangxi | Dominant | 72.74 ± 1.02d | 73.03 ± 1.06d | 6.39 ± 0.20d | 6.47 ± 0.44a | 3.58 ± 0.14d | 3.67 ± 0.21a | 17.29 ± 1.36a | 16.83 ± 0.94a | 0.2093 ± 0.0195a | 0.2025 ± 0.0135a |
| Guangxi | Co-dominant | 74.01 ± 1.05c | 75.04 ± 0.93c | 6.64 ± 0.22c | 6.25 ± 0.47ab | 3.76 ± 0.17c | 3.45 ± 0.24b | 15.58 ± 1.43b | 15.26 ± 0.91b | 0.1849 ± 0.0197b | 0.1802 ± 0.0126b |
| Guangxi | Intermediate | 75.34 ± 0.96b | 77.23 ± 0.84b | 6.93 ± 0.23b | 5.99 ± 0.52b | 3.97 ± 0.18b | 3.21 ± 0.24c | 13.76 ± 1.36c | 13.58 ± 0.83c | 0.1598 ± 0.0180c | 0.1572 ± 0.0111c |
| Guangxi | Suppressed | 76.48 ± 0.91a | 78.98 ± 0.83a | 7.20 ± 0.25a | 5.90 ± 0.87b | 4.18 ± 0.19a | 2.96 ± 0.27d | 12.13 ± 1.35d | 12.16 ± 0.70d | 0.1383 ± 0.0172d | 0.1384 ± 0.0090d |
| Sichuan | Dominant | 71.19 ± 1.89b | 73.32 ± 1.19a | 7.96 ± 1.02a | 6.83 ± 0.87a | 7.93 ± 0.45b | 6.90 ± 0.45d | 12.91 ± 0.74b | 12.95 ± 0.40d | 0.1484 ± 0.0096b | 0.1488 ± 0.0053d |
| Sichuan | Co-dominant | 72.29 ± 1.99ab | 73.37 ± 1.19a | 6.48 ± 0.96b | 5.63 ± 0.82b | 8.04 ± 0.48ab | 7.33 ± 0.46c | 13.18 ± 0.77b | 13.67 ± 0.37c | 0.1519 ± 0.0102b | 0.1583 ± 0.0050c |
| Sichuan | Intermediate | 73.08 ± 2.08a | 73.05 ± 1.22ab | 5.23 ± 0.87c | 4.63 ± 0.76c | 8.19 ± 0.53ab | 7.86 ± 0.52b | 13.50 ± 0.86ab | 14.46 ± 0.39b | 0.1562 ± 0.0115ab | 0.1690 ± 0.0053b |
| Sichuan | Suppressed | 73.63 ± 1.98a | 72.18 ± 1.19b | 4.02 ± 0.72d | 3.64 ± 0.63d | 8.39 ± 0.55a | 8.66 ± 0.60a | 13.96 ± 0.90a | 15.52 ± 0.46a | 0.1624 ± 0.0123a | 0.1838 ± 0.0064a |

*Data are presented as mean ± standard deviation. Different lowercase letters within each column indicate significant differences among dominance classes within a region (Duncan's test, P < 0.05). Abbreviations are defined in Table S1.*

**Table S3b. ANOVA P values supporting the density- and dominance-class trends shown in Figure 2.**

| **Allocation indicator** | **P_density** | **P_dominance** | **P_density × dominance** | **One-way P_dominance for Duncan grouping** |
| --- | --- | --- | --- | --- |
| SMF | 0.013047 | 8.1857e-09 | 1 | 5.1154e-09 |
| BMF | 0.067769 | 1.8957e-07 | 1 | 9.0506e-08 |
| LMF | 0.946452 | 0.999362 | 1 | 0.99927 |
| RMF | 0.055157 | 6.5774e-08 | 1 | 3.1308e-08 |
| R/S | 0.064691 | 5.2715e-08 | 1 | 2.3734e-08 |
| SCF | 0.08866 | 3.0609e-07 | 0.999997 | 1.4424e-07 |
| BCF | 7.5154e-04 | 7.9207e-15 | 0.999953 | 1.0344e-14 |
| LCF | 0.980258 | 0.975232 | 1 | 0.971737 |
| RCF | 0.705235 | 5.9872e-05 | 1 | 2.4720e-05 |
| C-R/S | 0.709751 | 5.4160e-05 | 1 | 2.2178e-05 |

*P_density, P_dominance, and P_density × dominance are from two-way ANOVA models with planting density, dominance class and their interaction as fixed factors. The one-way dominance P values provide the overall dominance-class ANOVA support for the Duncan multiple-range groupings reported in Table 2.*

**Table S4. Annual and seasonal ClimateAP variables used in sensitivity analyses.**

| **Region** | **CMD** | **MAT** | **MAP** | **DD5** | **Tmax_07** | **Tmax_sm** | **PPT_sp** | **PPT_sm** | **PPT_at** | **PPT_wt** | **CMD_at** | **CMD_sm** | **CMD_sp** | **CMD_wt** |
| --- | --- | --- | --- | --- | --- | --- | --- | --- | --- | --- | --- | --- | --- | --- |
| Fujian | 217.40 | 18.88 | 1831.15 | 5042.08 | 33.28 | 31.93 | 726.73 | 637.20 | 221.65 | 245.28 | 120.53 | 80.15 | 4.675 | 11.97 |
| Guangxi | 313.76 | 21.82 | 1312.65 | 6072.21 | 31.50 | 31.41 | 288.88 | 667.32 | 265.38 | 91.06 | 112.62 | 17.71 | 113.59 | 69.88 |
| Sichuan | 145.92 | 18.48 | 1147.38 | 4899.05 | 31.63 | 30.78 | 251.97 | 546.16 | 256.41 | 93.27 | 16.05 | 21.86 | 85.73 | 22.22 |

*MAT, mean annual temperature; MAP, mean annual precipitation; DD5, degree-days above 5 ℃; Tmax_07, July maximum temperature; Tmax_sm, summer maximum temperature; CMD, annual climatic moisture deficit; PPT_sp, PPT_sm, PPT_at and PPT_wt, spring, summer, autumn and winter precipitation, respectively; CMD_sp, CMD_sm, CMD_at and CMD_wt, spring, summer, autumn and winter climatic moisture deficit, respectively. Seasons are defined as spring (March–May), summer (June–August), autumn (September–November) and winter (December–February). Values in this table are unstandardized site-level climate values. The annual CMD values match Table S2; seasonal CMD variables were subsequently standardized across all observations before model fitting.*

**Table S5. Linear regression statistics for site-index relationships shown in Figures 3 and 4.**

| **Dominance class** | **n** | **slope** | **R^2^** | **P** | **allocation variable** |
| --- | --- | --- | --- | --- | --- |
| D | 45 | 0.0033 | 0.195 | 0.002 | SMF |
| CD | 45 | 0.0043 | 0.235 | <0.001 | SMF |
| I | 45 | 0.0055 | 0.291 | <0.001 | SMF |
| S | 45 | 0.0059 | 0.323 | <0.001 | SMF |
| D | 45 | -0.0016 | 0.153 | 0.008 | BMF |
| CD | 45 | -0.0017 | 0.161 | 0.006 | BMF |
| I | 45 | -0.0018 | 0.110 | 0.026 | BMF |
| S | 45 | -0.0011 | 0.034 | 0.229 | BMF |
| D | 45 | -0.0028 | 0.192 | 0.003 | LMF |
| CD | 45 | -0.0031 | 0.224 | 0.001 | LMF |
| I | 45 | -0.0036 | 0.273 | <0.001 | LMF |
| S | 45 | -0.0044 | 0.337 | <0.001 | LMF |
| D | 45 | 0.0012 | 0.030 | 0.252 | RMF |
| CD | 45 | 0.0005 | 0.012 | 0.472 | RMF |
| I | 45 | -0.0001 | 0.001 | 0.833 | RMF |
| S | 45 | -0.0003 | 0.005 | 0.646 | RMF |
| D | 45 | 0.0016 | 0.027 | 0.285 | R/S |
| CD | 45 | 0.0007 | 0.011 | 0.500 | R/S |
| I | 45 | -0.0002 | 0.001 | 0.814 | R/S |
| S | 45 | -0.0004 | 0.006 | 0.620 | R/S |
| D | 45 | 0.0013 | 0.080 | 0.059 | SCF |
| CD | 45 | 0.0028 | 0.207 | 0.002 | SCF |
| I | 45 | 0.0045 | 0.266 | <0.001 | SCF |
| S | 45 | 0.0055 | 0.232 | <0.001 | SCF |
| D | 45 | -0.0007 | 0.070 | 0.078 | BCF |
| CD | 45 | -0.0007 | 0.049 | 0.143 | BCF |
| I | 45 | -0.0006 | 0.028 | 0.268 | BCF |
| S | 45 | 0.0002 | 0.002 | 0.772 | BCF |
| D | 45 | -0.0020 | 0.178 | 0.004 | LCF |
| CD | 45 | -0.0024 | 0.172 | 0.005 | LCF |
| I | 45 | -0.0029 | 0.180 | 0.004 | LCF |
| S | 45 | -0.0039 | 0.207 | 0.002 | LCF |
| D | 45 | 0.0014 | 0.059 | 0.109 | RCF |
| CD | 45 | 0.0002 | 0.005 | 0.630 | RCF |
| I | 45 | -0.0010 | 0.135 | 0.013 | RCF |
| S | 45 | -0.0018 | 0.123 | 0.018 | RCF |
| D | 45 | 0.0019 | 0.054 | 0.123 | C-R/S |
| CD | 45 | 0.0003 | 0.005 | 0.642 | C-R/S |
| I | 45 | -0.0013 | 0.138 | 0.012 | C-R/S |
| S | 45 | -0.0025 | 0.129 | 0.015 | C-R/S |

*Abbreviations are defined in Table S1. Slopes are expressed in fraction units per 1 m increase in site index; multiplying by 100 gives percentage points per metre.*

**Table S6. Compact sensitivity summary comparing annual CMD, seasonal CMD and alternative climate-variable models.**

| **Response** | **model group** | **selected variable** | **AIC** | **marginal R^2^** | **climate main-effect direction** | **Singular fit** |
| --- | --- | --- | --- | --- | --- | --- |
| BCF | Annual CMD | CMD | -390.85 | 0.838 | negative | FALSE |
| BCF | Other climate variable | DD5 | -371.70 | 0.835 | positive | FALSE |
| BCF | Seasonal CMD | CMD_wt | -339.31 | 0.822 | positive | FALSE |
| BMF | Annual CMD | CMD | -375.74 | 0.895 | negative | FALSE |
| BMF | Other climate variable | MAT | -445.90 | 0.914 | negative | FALSE |
| BMF | Seasonal CMD | CMD_wt | -404.31 | 0.914 | negative | FALSE |
| LCF | Annual CMD | CMD | -580.08 | 0.970 | negative | FALSE |
| LCF | Other climate variable | PPT_sm | -677.82 | 0.974 | negative | FALSE |
| LCF | Seasonal CMD | CMD_at | -563.56 | 0.939 | negative | FALSE |
| LMF | Annual CMD | CMD | -384.90 | 0.944 | negative | FALSE |
| LMF | Other climate variable | PPT_at | -607.78 | 0.698 | negative | FALSE |
| LMF | Seasonal CMD | CMD_sp | -602.38 | 0.728 | negative | FALSE |
| RCF | Annual CMD | CMD | -747.29 | 0.820 | positive | FALSE |
| RCF | Other climate variable | PPT_sm | -746.51 | 0.820 | positive | FALSE |
| RCF | Seasonal CMD | CMD_at | -563.68 | 0.725 | positive | FALSE |
| RMF | Annual CMD | CMD | -660.92 | 0.674 | positive | FALSE |
| RMF | Other climate variable | DD5 | -606.56 | 0.651 | positive | FALSE |
| RMF | Seasonal CMD | CMD_wt | -523.49 | 0.603 | positive | FALSE |
| RS | Annual CMD | CMD | -613.76 | 0.680 | positive | FALSE |
| RS | Other climate variable | DD5 | -557.53 | 0.657 | positive | FALSE |
| RS | Seasonal CMD | CMD_wt | -471.99 | 0.610 | positive | FALSE |
| RSC | Annual CMD | CMD | -695.44 | 0.822 | positive | FALSE |
| RSC | Other climate variable | PPT_sm | -691.64 | 0.821 | positive | FALSE |
| RSC | Seasonal CMD | CMD_at | -507.76 | 0.726 | positive | FALSE |
| SCF | Annual CMD | CMD | -1144.42 | 0.894 | negative | FALSE |
| SCF | Other climate variable | PPT_sm | -1175.23 | 0.906 | negative | FALSE |
| SCF | Seasonal CMD | CMD_at | -1104.38 | 0.889 | negative | FALSE |
| SMF | Annual CMD | CMD | -1116.31 | 0.851 | positive | FALSE |
| SMF | Other climate variable | Tmax_sm | -1150.28 | 0.869 | positive | FALSE |
| SMF | Seasonal CMD | CMD_at | -1136.55 | 0.861 | positive | FALSE |

*Each model retained the main fixed-effect structure used in the revised manuscript and replaced annual CMD with one alternative climate variable at a time. The model group with the lowest AIC within each climate set is shown. Abbreviations are defined in Table S1 and Table S4. Because dominance class was treated as a categorical factor with dominant trees as the reference level and climate × dominance-class interactions were included, the climate main-effect direction refers to the direction of the climate coefficient for dominant trees, not to an overall class-averaged effect. Suppressed-tree climate slopes are reported separately in Table S7.*

**Table S7. Suppressed-tree slopes for annual and seasonal CMD in key allocation variables.**

| **Response** | **Climate variable** | **climate slope** | **SE** | **Wald P** | **slope direction** | **AIC** | **delta AIC vs annual CMD** | **marginal R^2^** | **LRT P** | **Singular fit** |
| --- | --- | --- | --- | --- | --- | --- | --- | --- | --- | --- |
| RCF | CMD | -0.0941 | 0.0062 | <0.001 | negative | -747.29 | 0.00 | 0.820 | <0.001 | FALSE |
| RCF | CMD_at | -0.0974 | 0.0101 | <0.001 | negative | -563.68 | 183.61 | 0.725 | <0.001 | FALSE |
| RCF | CMD_wt | -0.0805 | 0.0089 | <0.001 | negative | -505.54 | 241.75 | 0.657 | <0.001 | FALSE |
| RCF | CMD_sp | -0.0449 | 0.0162 | 0.006 | negative | -377.57 | 369.72 | 0.349 | <0.001 | TRUE |
| RCF | CMD_sm | 0.0218 | 0.0200 | 0.274 | positive | -356.89 | 390.40 | 0.270 | 0.071 | TRUE |
| RMF | CMD | -0.0598 | 0.0099 | <0.001 | negative | -660.92 | 0.00 | 0.674 | <0.001 | FALSE |
| RMF | CMD_wt | -0.0556 | 0.0115 | <0.001 | negative | -523.49 | 137.43 | 0.603 | <0.001 | FALSE |
| RMF | CMD_at | -0.0383 | 0.0150 | 0.011 | negative | -464.39 | 196.53 | 0.577 | <0.001 | FALSE |
| RMF | CMD_sp | -0.0302 | 0.0208 | 0.146 | negative | -370.68 | 290.24 | 0.393 | <0.001 | FALSE |
| RMF | CMD_sm | 0.0190 | 0.0269 | 0.481 | positive | -340.14 | 320.79 | 0.312 | <0.001 | FALSE |
| SCF | CMD | 0.0313 | 0.0017 | <0.001 | positive | -1144.42 | 0.00 | 0.894 | <0.001 | FALSE |
| SCF | CMD_at | 0.0388 | 0.0024 | <0.001 | positive | -1104.38 | 40.03 | 0.889 | <0.001 | FALSE |
| SCF | CMD_wt | 0.0284 | 0.0023 | <0.001 | positive | -1013.56 | 130.85 | 0.830 | <0.001 | FALSE |
| SCF | CMD_sp | 0.0248 | 0.0043 | <0.001 | positive | -906.06 | 238.35 | 0.712 | <0.001 | FALSE |
| SCF | CMD_sm | -0.0188 | 0.0057 | <0.001 | negative | -884.13 | 260.29 | 0.667 | 0.028 | FALSE |
| SMF | CMD | 0.0078 | 0.0024 | 0.001 | positive | -1116.31 | 0.00 | 0.851 | 0.003 | FALSE |
| SMF | CMD_at | 0.0127 | 0.0032 | <0.001 | positive | -1136.55 | -20.24 | 0.861 | <0.001 | FALSE |
| SMF | CMD_sm | 0.0017 | 0.0054 | 0.748 | positive | -1124.55 | -8.24 | 0.840 | <0.001 | FALSE |
| SMF | CMD_sp | 0.0025 | 0.0043 | 0.563 | positive | -1117.96 | -1.65 | 0.839 | 0.001 | FALSE |
| SMF | CMD_wt | 0.0060 | 0.0026 | 0.023 | positive | -1110.40 | 5.90 | 0.845 | 0.038 | FALSE |

*Abbreviations are defined in Table S1 and Table S4. Delta AIC versus annual CMD was calculated as AIC (seasonal CMD model) − AIC (annual CMD model) for the same response variable. Negative values indicate that the seasonal CMD model had a lower AIC than the corresponding annual CMD model, whereas positive values indicate a higher AIC.*

**Table S8. Sensitivity of annual CMD main-effect direction after adding one age, geographic/topographic, or tree-size covariate at a time.**

| **Response** | **tested covariates (n)** | **same CMD direction (n)** | **changed-direction covariates** | **base marginal R^2^** | **median adjusted marginal R^2^** | **singular models (n)** |
| --- | --- | --- | --- | --- | --- | --- |
| BCF | 8 | 5 | Mean_DBH; Mean_H; H | 0.838 | 0.842 | 0 |
| BMF | 8 | 8 | none | 0.895 | 0.915 | 0 |
| LCF | 8 | 8 | none | 0.970 | 0.973 | 0 |
| LMF | 8 | 8 | none | 0.944 | 0.965 | 0 |
| RCF | 8 | 8 | none | 0.820 | 0.820 | 0 |
| RMF | 8 | 8 | none | 0.674 | 0.684 | 0 |
| R/S | 8 | 8 | none | 0.680 | 0.690 | 0 |
| C-R/S | 8 | 8 | none | 0.822 | 0.822 | 0 |
| SCF | 8 | 6 | Age; Latitude | 0.894 | 0.902 | 0 |
| SMF | 8 | 7 | Mean_H | 0.851 | 0.865 | 0 |

**Table S9. Sensitivity of the suppressed-tree annual CMD slope after adding one age, geographic/topographic, or tree-size covariate at a time.**

| **Response** | **tested covariates (n)** | **same suppressed CMD-slope direction (n)** | **changed suppressed CMD-slope covariates** | **base suppressed CMD slope** | **median adjusted suppressed CMD slope** | **singular models (n)** |
| --- | --- | --- | --- | --- | --- | --- |
| RCF | 8 | 8 | none | -0.0941 | -0.0940 | 0 |
| RMF | 8 | 7 | Latitude | -0.0598 | -0.0564 | 0 |
| SCF | 8 | 8 | none | 0.0313 | 0.0324 | 0 |
| SMF | 8 | 8 | none | 0.0078 | 0.0093 | 0 |

*Notes for Tables S8 and S9: Sensitivity analyses were interpreted as robustness checks for site-level associations, not as independent causal tests. Age, stand age; Elevation, Latitude and Longitude, site-level geographic/topographic variables; Mean_DBH and Mean_H, site-level mean diameter at breast height and mean tree height; DBH and H, dominance-class-level mean diameter at breast height and mean tree height. Other abbreviations are defined in Table S1.*


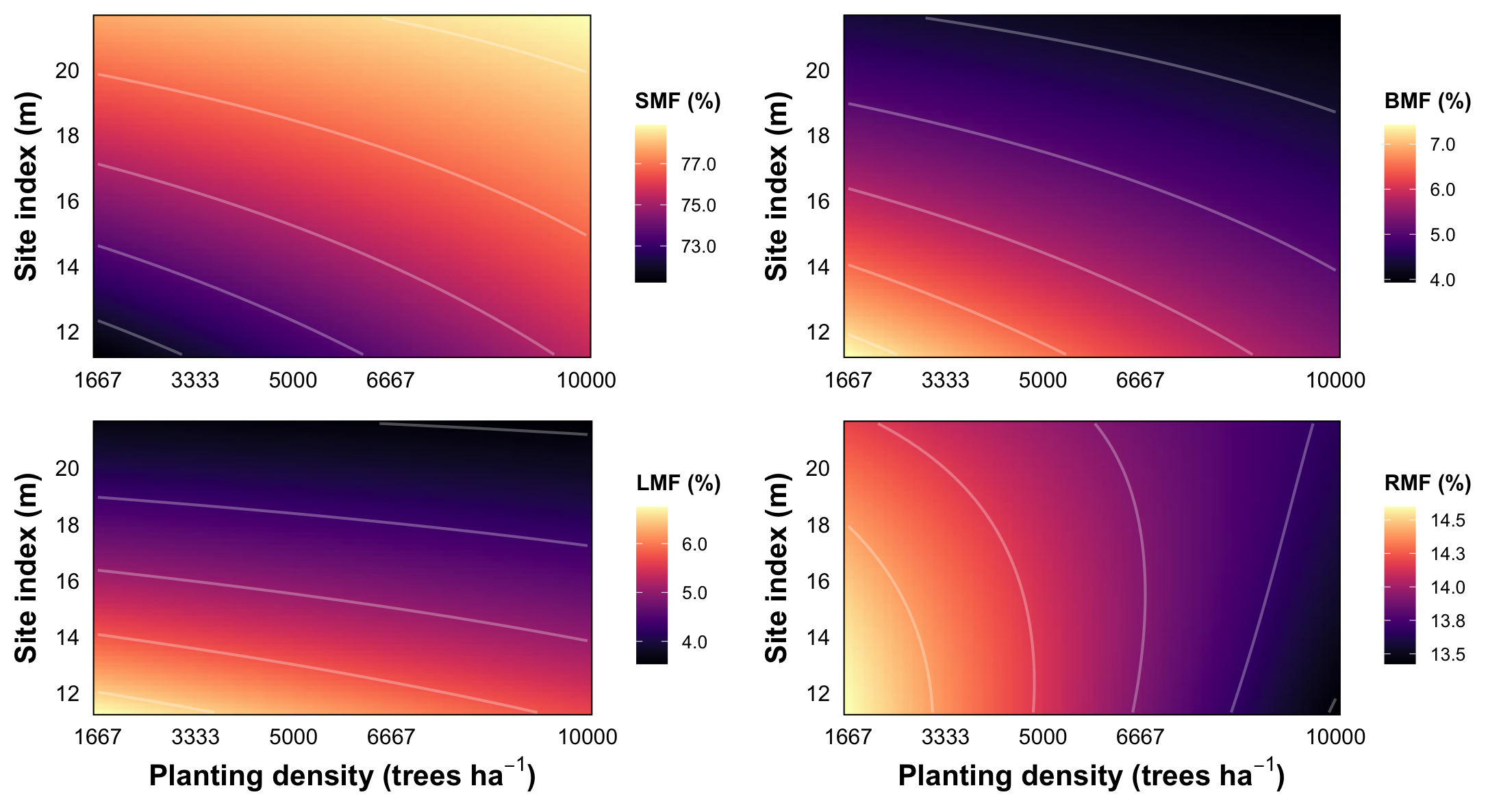


**Figure S1. Interactive effects of planting density and site index on biomass allocation fractions in different dominance classes.**


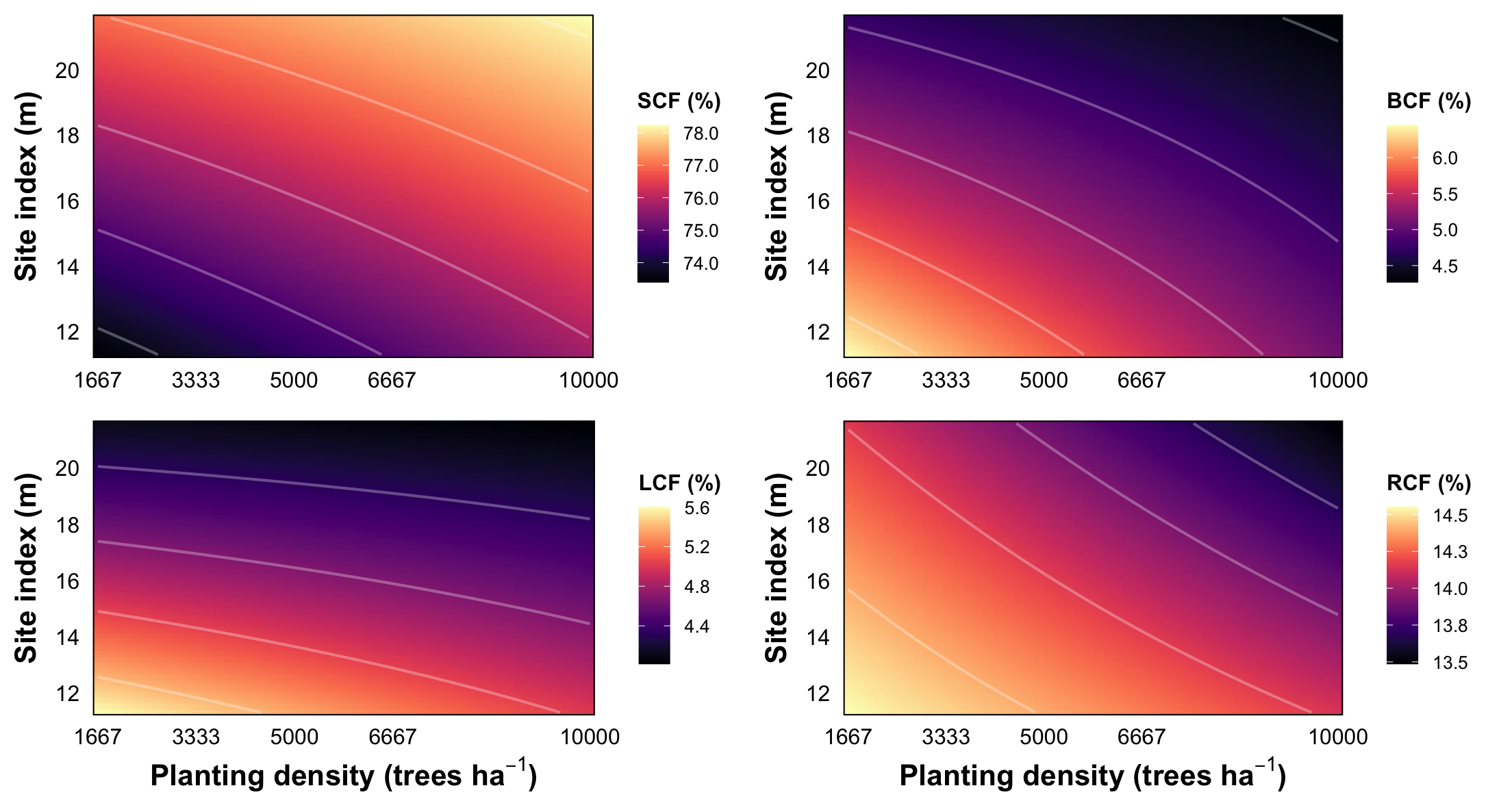


**Figure S2. Interactive effects of planting density and site index on carbon allocation fractions in different dominance classes.**

**Figure S3. Variation in biomass allocation fractions (BAFs) and carbon allocation fractions (CAFs) of Chinese fir across initial planting densities for different dominance classes.** Within each boxplot, the box represents the interquartile range (IQR, 25th–75th percentile). The whiskers extend to the most extreme values within 1.5 × IQR from the quartiles, and values beyond this range were treated as outliers and not displayed. Colors indicate the gradient of initial planting density. Abbreviations: SMF, stem biomass fraction; BMF, branch biomass fraction; LMF, leaf biomass fraction; RMF, root biomass fraction; SCF, stem carbon fraction; BCF, branch carbon fraction; LCF, leaf carbon fraction; RCF, root carbon fraction; R/S, root-to-shoot ratio of biomass; C-R/S, root-to-shoot ratio of carbon; D, dominant trees; CD, co-dominant trees; I, intermediate trees; S, suppressed trees.
